# Management of an Oral Ingestion of Transdermal Fentanyl Patches: A Case Report and Literature Review

**DOI:** 10.1155/2011/495938

**Published:** 2011-05-12

**Authors:** Andrew C. Faust, Ralph Terpolilli, Darrel W. Hughes

**Affiliations:** ^1^Department of Pharmacy Services, University Health System, 4502 Medical Drive, San Antonio, TX 78229, USA; ^2^Pharmacotherapy Division, College of Pharmacy, The University of Texas at Austin, Austin, TX 78712, USA; ^3^Pharmacotherapy Education and Research Center, The University of Texas Health Science Center at San Antonio, San Antonio, TX 78229, USA; ^4^Division of Emergency Medicine, The University of Texas Health Science Center at San Antonio, San Antonio, TX 78229, USA

## Abstract

*Purpose*. Fentanyl is available as a transdermal system for the treatment of chronic pain in opioid-tolerant patients; however, it carries a black box warning due to both the potency of the product and the potential for abuse. In this report, we describe a case of transbuccal and gastrointestinal ingestion of fentanyl patches and the management of such ingestion. *Summary*. A 32-year-old man was brought to the emergency department (ED) via emergency medical services for toxic ingestion and suicide attempt. The patient chewed and ingested two illegally purchased transdermal fentanyl patches. In the ED, the patient was obtunded, dizzy and drowsy. Initial vital signs showed the patient to be afebrile and normotensive with a heart rate of 63, respiratory rate of 16, and oxygen saturation of 100% on 2 liters nasal cannula after administration of 2 milligrams of intravenous naloxone. The patient was treated with whole bowel irrigation and continuous intravenous naloxone infusion for approximately 48 hours without complications. *Conclusion*. Despite numerous case reports describing oral ingestion of fentanyl patches, information on the management of such intoxication is lacking. We report successful management of such a case utilizing whole bowel irrigation along with intravenous push and continuous infusion naloxone.

## 1. Introduction

Fentanyl, a potent phenylpiperidine opioid agonist available as a transdermal patch, carries a black box warning cautioning health care providers on both the potency of the product and the potential for abuse and diversion [1]. Numerous consumer Internet websites describe means for abusing fentanyl patches, such as smoking, chewing, freezing, or intravenous injection of the gel reservoir [2, 3]. In 2005, Goldberger and colleagues presented data from the state of Florida which found fentanyl as the cause of 115 deaths [4]. Although anecdotal reports exist, there is little published in medical literature regarding oral intoxication of fentanyl patches [5–8]. We describe both the presentation and management of a patient who ingested two fentanyl patches.

## 2. Case Report

A 32-year-old white male was brought to the emergency department (ED) via emergency medical services (EMS) for a toxic ingestion. The patient was in a verbal altercation around 0700 with his ex-wife, resulting her calling the police department. After discovering law enforcement was en route, his ex-wife witnessed the patient chewing and ingesting two illegally purchased 50 micrograms per hour transdermal fentanyl patches, along with 6–8 milligrams of alprazolam. EMS found the patient to be nonresponsive to verbal or physical stimuli, bradycardic, and with miotic pupils. Two milligrams of intravenous naloxone were administered by EMS, which resulted in opioid reversal and arousal of the patient. The patient was subsequently transferred to our ED with findings of altered mental status, obtundation, and drowsiness. On arrival, law enforcement corroborated the ex-wife's description of the patient ingesting the two fentanyl patches and gave an account of the event to the ED physician and pharmacists. The patient admitted to regular use of methamphetamine, opioids, and benzodiazepines and had a past medical history bipolar disorder for which he took no medications. His review of systems was negative, except for the dizziness, drowsiness, and altered mental status, and admission physical examination was unremarkable. The patient's initial set of vitals after 2 milligrams of intravenous naloxone were as follows: afebrile, blood pressure 106/77, heart rate 63, respiratory rate 16, and oxygen saturation 100% on 2 liters via nasal cannula. Initial chemistry, liver function tests, and complete blood count were all within normal limits. A urine drug screen was negative for opioids, but positive for amphetamines and benzodiazepines. Serum ethanol, acetaminophen, and salicylate levels were all negative. A serum fentanyl level was not obtained. 

The patient arrived in the ED at 0900. Upon arrival, the patient was stabilized and assessed. At 1000, a naloxone drip (8 micrograms per milliliter in 0.9% sodium chloride) was initiated at 0.4 milligrams per hour and slowly titrated up to 2 milligrams per hour due to returning signs of opioid toxicity (bradycardia, respiratory rate <12, and declining neurologic exam). Additionally, whole bowel irrigation with polyethylene glycol 3350 via nasogastric tube was initiated secondary to concerns for ingested patches creating a depot-like effect. The patient had one bowel movement in the ED (no documentation that patches or remnants were passed) and remained hemodynamically and neurologically stable until transfer to the medical intensive care unit at approximately 2200 that evening. His naloxone drip was titrated down to 0.2 milligrams per hour around 1200 of his second hospital day and was eventually discontinued that evening at 2300. He did not receive any additional naloxone after this point. In total, he received approximately 15 milligrams of naloxone over these two days. He remained cardiovascularly and neurologically stable throughout his stay in the medical ICU, was subsequently downgraded to the medical floor on hospital day 3 (48 hours after admission), and discharged from the hospital on day 4 at 1100. 

## 3. Discussion

Multiple ingestions of fentanyl patches have been reported, with outcomes ranging from transient symptoms of overdose (e.g., hypotension, altered mental status) to death. Van Rijswijk and Van Guldener described the case of an 81-year-old male with a history of multiple myeloma, osteoporosis, and delirium who was found chewing on a fentanyl patch [5]. His nurse promptly removed the patch from the patient's mouth; however, thirty minutes later, the patient became less arousable and hypotensive. After administration of naloxone 0.4 milligrams and intravenous saline, the patient's mental status and blood pressure returned to baseline. Thomas and colleagues published a case report of a 42-year-old male with known drug abuse, including hospitalization for drug overdose, who died subsequent to oral ingestion of fentanyl patches [6]. Upon autopsy, the decedent had three pieces of fentanyl patches in his stomach. Further history reveals that the decedent commonly chewed, sucked on, and swallowed fentanyl patches to obtain a high. Lastly, Woodall and colleagues reported a case series of seven deceased patients in whom oral administration of a fentanyl patch was suspected to have contributed to their death [7]. Decedents ranged in age from 20 to 51 years and had oral and transbuccal administration of a patch based on witness reports or evidence of patch residue in the oral cavity. Postmortem fentanyl serum concentrations ranged from 7 to 97 ng/mL, whereas typical serum concentrations from single dose studies with fentanyl transbuccal tablets (100–800 mcg) yield peak serum concentrations between 0.25 and 1.59 ng/mL [9]. More recently, Carson and colleagues presented a case of a 28-year-old white male who died in the ED after chewing and aspirating a transdermal fentanyl patch. On autopsy, the decedent had a beige foreign body (later identified as the transdermal device) lodged in the mainstem bronchus, and postmortem toxicological analysis revealed a serum fentanyl level of 8.6 ng/mL [8]. Fentanyl serum levels are not performed in our facility's laboratory, and determining them would require sending them to an outside laboratory. Due to the typical return time on these types of labs, we felt an admission fentanyl level would be of little benefit to the medical team three days later.

Fentanyl is 50–65% bioavailable across the buccal membrane when administered as a transbuccal system (e.g., lozenge and troche) [1, 9, 10]. However, when ingested orally, fentanyl undergoes extensive first-pass metabolism, resulting in 20% escaping hepatic metabolism and entering the systemic circulation. Therefore, as alluded to in published case reports, the time fentanyl stays in contact with the oral mucosa directly translates to the systemic absorption and the severity of the overdose. Case reports describe either chewing or sucking on the fentanyl patch, leading to extensive contact time between the oral mucosa and the inner gel matrix. Additionally, the reservoir of a fentanyl patch houses a large dose of fentanyl. In the case of our patient, a 50 microgram per hour patch stores 8400 micrograms of fentanyl [1]. If the entire reservoir contents of one fentanyl patch were ingested, approximately 1680 micrograms would enter the systemic circulation. Assuming a 25% reduction for cross-tolerance, this fentanyl dose is equivalent to 126 milligrams of intravenous morphine [11]. However, ingesting an intact patch would not necessarily result in significant gastrointestinal absorption (i.e., the drug would still remain within the intact system). If the entire contents of one patch were removed and administered transbuccally, this systemic level would be approximately three times that achieved with gastrointestinal absorption (i.e., approximately 5000 micrograms of fentanyl or 375 milligrams of intravenous morphine). Thus, chewing or sucking on a fentanyl patch increases contact time with the buccal membrane and liberates drug from the transdermal system, greatly increasing systemic absorption.

Our patient's ED urine drug screen for opioids was negative; however, this does not necessarily exclude an opioid ingestion. A urine drug screen's ability to detect substances is based on the assay, the drug (and metabolites, if applicable) the screen is designed for, and a drug's pharmacokinetics [12]. To be detected in a urine drug screen, the substance must be absorbed, metabolized, and then renally excreted. In general, most standard urine screens for opioids detect codeine, morphine, and metabolites of the two. Fentanyl is a fully synthetic opiate that is structurally dissimilar from morphine and codeine; therefore, the metabolites of fentanyl are also dissimilar [12]. Fentanyl is primarily hepatically metabolized, and the inactive metabolites (primarily norfentanyl) are excreted in the urine. Only 10% of the parent drug is excreted unchanged in the urine [11]. In order to detect a fentanyl-related opioid intoxication, a urine drug screen would need to assay exclusively for fentanyl or the predominant metabolite norfentanyl. 

The treatment approach in this patient was twofold: limit opioid toxicity and facilitate passage of the ingested patches. Naloxone, a pure opioid antagonist at all opioid receptor sites, has long been utilized in the ED and intensive care unit for the treatment of opioid intoxication [11]. Whole bowel irrigation using polyethylene glycol or a similar osmotic laxative has been used successfully in the treatment of other intoxications, such as lithium or ingestion of extended release products [13, 14]. While activated charcoal is often a first choice for oral intoxications, we chose to utilize a different approach for two reasons. First, the efficacy of single-dose-activated charcoal decreases over time. In a 2005 position paper, Chyka and Seger demonstrated decreased efficacy as the time from poisoning is increased from 30 minutes postingestion to 180 minutes [15]. These authors questioned the utility of charcoal use after one hour postingestion. Secondly, drug must be available in the gastrointestinal tract and must be bound by charcoal for this treatment modality to be effective [16]. Since the time from ingestion to presentation to our ED was over 2 hours, the drug was, at least partially, housed within the transdermal system, and there are no data on adsorption of fentanyl to activated charcoal, we opted away from this therapeutic option. Our approach was to utilize a continuous infusion of naloxone until the patient passed the patches in his stool and returned to his baseline mental status. Unfortunately, the patches were never recovered in the patient's stool and were later presumed to have passed prior to transfer to the intensive care unit, although it is possible that the patient extracted the fentanyl from the patches and disposed them in a conventional means prior to law enforcement and EMS arrival. Fentanyl absorbed across the buccal membrane has an elimination half-life of up to 12 hours; therefore, treating and monitoring the patient for 72 hours would ensure, using the most conservative estimate, 6 half-lives had passed and at least 98.5% of the drug had been eliminated [10].

Lastly, the patient coingested 6–8 milligrams of alprazolam, perhaps leading to benzodiazepine overdose. While a specific antidote for benzodiazepine ingestion exists (i.e., Flumazenil), we did not utilize it because of the unknown duration of alprazolam (or other benzodiazepine) use. Flumazenil may precipitate benzodiazepine withdrawal or seizures, and routine administration of Flumazenil has been questioned, leading some to recommend that the drug rarely be utilized in benzodiazepine overdose [17, 18]. Additionally, despite an 11-hour half-life, the duration of action of alprazolam is 5.1 ± 1.7 hours [11]. We, therefore, adopted a more conservative approach when treating this benzodiazepine ingestion by providing supportive care and whole bowel irrigation.

## 4. Conclusions

Our case adds to the small body of literature which describes oral ingestion of a fentanyl patch and is the only to describe a treatment modality for this situation. Contact time with the oral mucosa may be the most important factor in determining severity of oral fentanyl ingestion, as bioavailability is significantly higher via the buccal membrane.
